# Some Tools and Methodologies for Domain Ontology Building

**DOI:** 10.1002/cfg.242

**Published:** 2003-02

**Authors:** Aldo Gangemi

**Affiliations:** 1 Laboratory for Applied Ontology Institute of Cognitive Sciences and Technology National Research Council (ISTC-CNR) Viale Marx 15 Rome 00137 Italy


**Comparative and Functional Genomics**
*Comp Funct Genom* 2003; **4**: 104–110.
Published online in Wiley InterScience (www.interscience.wiley.com). **DOI:** 10.1002/cfg.242

**Conference Review**

### Some tools and methodologies for domain ontology building

Aldo Gangemi*
*Laboratory for Applied Ontology, Institute of Cognitive Sciences and Technology, National Research Council (ISTC-CNR), Viale Marx 15, 00137 Rome, Italy*

**Correspondence to: Aldo Gangemi, Laboratory for Applied Ontology, Institute of Cognitive Sciences and Technology, National Research Council (ISTC-CNR), Viale Marx 15, 00137 Rome, Italy. E-mail: gangemi@ip.rm.cnr.it, http://ontology.ip.rm.cnr.it*

Received: 5 November 2002
Accepted: 29 November 2002

### Overview

The Laboratory for Applied Ontology (OntoLab) is a distributed structure (Rome and Trento) of the Institute of Cognitive Sciences and Technology (a section of the Italian National Research Council). It performs basic and applied research on the ontological foundations of conceptual modelling, exploring the role of ontologies in different fields, such as knowledge representation, knowledge engineering, database design, information retrieval, natural language processing and the semantic web.

OntoLab is coordinated by Nicola Guarino, and currently employs five full-time research scientists (besides the author and the coordinator): S. Borgo, C. Masolo, A. Oltramari, D.M. Pisanelli and G. Steve. The group is characterized by an interdisciplinary approach that combines computer science, philosophy and linguistics and relies on logic as a unifying paradigm.

Although its core interest is in methodologies and theories, the research of OntoLab addresses, or makes use of, artifacts developed in all the basic areas of ontology engineering:

- Logical languages to represent ontologies.
- Computational issues to reason with ontological knowledge.
- Methodologies for building, analysing and merging ontologies.
- Tools that support methodologies.
- Ontological theories.
- Linguistic processing for ontology lexicalization.
- Languages and tools that interface ontological theories with other applications.

### Projects and programs

Current projects at OntoLab include 5thFP WonderWeb, in which we are developing a library of *foundational* (i.e. domain-independent) ontologies for the semantic web [10] and the thematic network OntoWeb, especially as far as the Content Standard Harmonization SIG is concerned [9]. Another project is EUREKA Intelligent Knowledge Fusion (IKF), with enterprise consulting services on ontologies and assistance to software houses in developing innovative applications in banking [5], insurance, and service-level management domains. The UN-FAO joint project Fishery

Copyright © 2003 John Wiley & Sons, Ltd.

Ontology Service (FOS) is dedicated to the merging of several fishery terminologies, in order to support ontology-based information retrieval and other web services [6].

Some long-term research programs were initiated several years ago that apply conceptual methodologies and tools to the following domains in order to develop *core* (domain-generic) ontologies: law (harmonization of existing core ontologies); biomedicine (analysing and merging terminologies); and planning (developing a novel core ontology for plans, guidelines, etc.).

Another medium-term program is dedicated to the analysis and refinement of the WordNet lexical repository, an activity carried out in collaboration with the University of Princeton team that developed WordNet [4].

### Conceptual tools

OntoLab produces conceptual tools and methodologies to build and maintain domain ontologies whose quality can be assessed against explicit criteria. A domain ontology, according to our qualitative standards, is an axiomatic theory containing concepts and relations that can play the role of *generic references* for the intended meaning of the terms used by a community, being as accurate and explicit as possible.

Our tools provide explicit criteria to classify concepts and relations, and to allow different people that work in different areas to have an intuition of what someone has put in his/her ontology.

For example, a domain ontology in biology may contain definitions of ‘species’, ‘organism’, ‘pathway’, ‘anatomical structure’, ‘biological process’, etc. Our tools help the encoder of the ontology to decide whether his/her meaning of ‘species’ is about organisms or classes of organisms; whether the meaning of ‘function’ is about substances or processes involving substances; and whether the meaning of ‘pathway’ denotes real biological processes or theoretical reconstructions of processes, etc. A user of that ontology (or a software agent using it) will then be aware of the encoder’s meaning on a transparent basis.

The main tools and methodologies currently available from OntoLab are:

- The Descriptive Ontology for Linguistic and Cognitive Engineering (DOLCE) foundational ontology [10]. This is the first module of a future Library of Foundational Ontologies. A foundational ontology contains a description of the basic kinds of entities and relationships that are assumed to exist in some domain, such as *process, object, time, part, location, representation*, etc. (Figure 1). DOLCE is a cognitively-oriented ontology, based on primitive space and time,

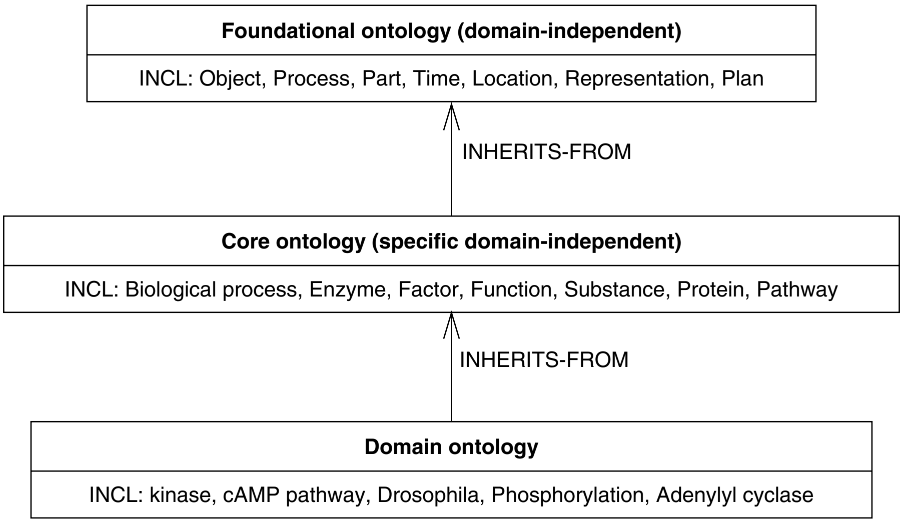

**Figure 1.** A simplified example of an ontology library: a *foundational* ontology contains domain-independent ontology elements; a *core* biological ontology contains general elements for a domain; a *domain* biological ontology contains the ontology elements needed for a domain to be conceptualized according to some task

Copyright © 2003 John Wiley & Sons, Ltd.

three-dimensional intuition (objects are disjoint from processes), and distinction between physical and intentional objects, etc. DOLCE is a *descriptive* ontology because it helps with categorizing a previously formed conceptualization: it does not state how things *are* but how they can be represented, according to some existing knowledge.

- The OntoClean methodology and meta-properties [8]. Currently implemented in most toolkits for ontology development, this provides a means of remodelling existing ontologies by separating their backbone, a stable taxonomy, from accessory hierarchies.
- The Ontological Integration of Naïve Sources (ONIONS) methodology [2]. This provides guidelines to analyse and merge existing ontologies and emphasizes the re-use of domain terminologies. This is described in more detail below.
- The OnionLeaves library. This is a library containing plug-ins (so-called *core axiom schemata*) to the DOLCE foundational ontology [3]. Currently, it includes plug-ins for *plans, communication, spatial location* relations and *functional participation* relations.

### Methodological approaches and ONIONS

Three main methodology types for ontology construction can be singled out from the literature. The first (*community ontology*) assumes neither foundational nor core ontologies, but tries to negotiate an intersubjective agreement among the members of a community of interest. The second (*linguistic ontology*) deals with a lexicographic treatment of domain terminologies, usually in an informal way (most dictionaries and thesauri can be considered as products of this activity). The third (*cognitive ontology*) calls for axiomatic theories and philosophical notions to be used in performing domain analysis.

ONIONS is a methodology for conceptual analysis and merging of terminologies. It integrates the three kinds of methodology by taking into account the results of linguistic ontology, and applying to them the techniques of cognitive ontology to reach an intersubjective agreement with the help of domain experts. An *intersubjective agreement* here is an agreement that applies to different contexts of the use of a terminology.

ONIONS has been under development since 1993. During this time, it has been applied to the construction of a medical core ontology (ON9 [2]), to an ontological mining of the UMLS™ repository [2] and to the integration of clinical guideline standards [11]. More recently, it has been applied to web catalogues, legal regulations [5], banking procedures, a revisitation of WordNet [4], and merging fishery terminologies [6], etc.

ONIONS aims to provide extensive axiomatization and ontological depth to the domain terminologies that are to be analysed, integrated or merged.

Axiomatization is obtained through the conceptual analysis of the terminological sources and their representation in a logical language. The different logical theories then need to be integrated. Logical integration in ONIONS assumes the Ontology Integration Framework [1], which describes the construction of a unified theory containing the union of the names and axiom sets from the sources, and the definition of *mapping relations* that allow unified queries to the sources.

Ontological depth of the analysis is obtained by re-using a library of foundational ontologies, on which the axiomatization depends. Such a library may include multiple choices among partially incompatible ontologies.

Very briefly, terminological analysis in ONIONS is carried out as shown in Table 1. Once individual terminologies have been updated, they can be *merged* by following additional guidelines [2,6]. The most important tools for performing merging in ONIONS are so-called '*core axiom schemata*'.

### Core axiom schemata

To conclude this short review of some domain ontology building techniques developed by OntoLab, an example of a *core axiom schema* is provided, for domains dealing with descriptions and situations (Figure 2). This has been specialized in order to build core ontologies in several application domains: clinical guidelines [11], banking regulations and legal norms [5], fishing techniques, service-level management, etc.

According to the schema, the components of a *situation* are *perdurants* (≈activities, processes or events), *endurants* (≈objects) that participate

Copyright © 2003 John Wiley & Sons, Ltd. 

**Table 1.** ONIONS life cycle for domain analysis

| Steps | Activities | Example |
|---|---|---|
| 1. | *Terminological source collection* | UMLS sources, scientific articles, etc. To demonstrate the principle, a sample term is singled out, e.g. *viral hepatitis, type B* |
| 2. | *Ontology architecture design*. The application domain that the sources are about is investigated in order to create a preliminary graph of ontology modules (*ontology library*) and context types (*context typology*) | Graph including at least some ontologies (besides a foundational one): anatomy, morphology, functions, diagnosis (cf. SNOMED nomenclature)<br>The context types for a certain task (clinical, epidemiological, research, administrative . . .) |
| 3. | *Source analysis*. All data from any source that are relevant for conceptual analysis (terms, lexical relations, synonyms, etc.) are collected and generically described | Taxonomies including *inflammation*-related knowledge, and how they are structured |
| 4. | *Term extraction*. When data are structured in a plain text, relevant domain terms have to be extracted according to available heuristics | *Inflammation*-related terms extracted from free text. |
| 5. | *Data are imported and translated* to a common format. | Taxonomies and other data on *inflammations* are wrapped into a logical format |
| 6. | *Core ontology formation*. Additional ontologies can be reused according to the preliminary ontology library, and the requirements that come from source analysis. Usually these ontologies are *merged* in order to build a preliminary *core* ontology for the domain. ONIONS merging guidelines are described in Gangemi *et al.* 1999 [2] | A core ontology for *inflammation* according to different contexts is built, e.g. it contains concepts for *inflammation conditions, inflammation processes, inflamed areas, inflammation morphologies, inflammation diagnoses, clinical costs for inflammation, inflammation aetiologies, symptomatic forms, detected antigens*, etc., and the relations between them |
| 6.1. | If no existing ontology can be retrieved, or if they are inadequate to build the core ontology, some additional ontology elicitation is performed from experts and basic manuals | The development of some parts of the *inflammation* core ontology has needed experts' advice. |
| 6.2. | The core ontology should be integrated with a *foundational ontology*, providing a set of domain-independent criteria that are capable of justifying the conceptual structure of the domain | The *inflammation* core ontology has been built according to DOLCE concepts and relations [10] |
| 6.3. | The basic ingredients of a core ontology are the so-called *core axiom schemata*, that formalize the dependencies among the basic concept and relations used in a domain application | Systematic relations between some of the multiple meanings for *inflammation* are used to build a *core axiom schema*. Such a schema is used to model all kinds of inflammations from the sources. An example of the schema is provided in Figure 2 |
| 7. | *Terminological analysis*. The terms found in the sources receive *plain text descriptions* if not available, and they are analysed according to *linguistic rules*. Plain text descriptions are built according to an accessory set of guidelines | Analysis of sample term: *viral hepatitis type B*:<br>* descriptions: 'inflammation of the liver', 'an acute illness caused by hepatitis B virus', etc.<br>* dependency rules: *inflammation* ⇒ *liver*, *illness* ⇒ *acute*, *virus* ⇒ *hepatitis*, *hepatitis* ⇒ *B*, etc. |
| 8. | *Ontology data type assignment*. Once all the terms (or other useful data) from sources and descriptions have been analysed, the resulting components are assigned to some *ontology data type*: individual, concept, relation, etc. Concepts can also receive a metaproperty assignment [8] | inflammation ∈ concept (*rigid*)<br>liver ∈ concept (*rigid*)<br>virus ∈ concept (*rigid*)<br>acute ∈ concept (*non-rigid*)<br>caused by ∈ relation<br>patient#John_Hepaticus ∈ individual, etc. |

Copyright © 2003 John Wiley & Sons, Ltd.

**Table 1.** Continued

| Steps | Activities | Example |
|---|---|---|
| 9. | *Vertical integration*. Ontology data are integrated with the core ontology. The core ontology provides the concepts and relations that subsume those assigned in 8 | *inflammation* → {diagnosis\|condition\|process\|*morphology*\|anatomical *feature*}liver → organ → body part → biological object*HBVirus* → organism → biological object*acute* → condition quality → quality*causedby* → depends on, etc. |
| 10. | *Formalization*. The ontology data obtained are put into a logical form and checked for consistency. If terminological analysis is carried out with an ontology editor that implements a logical language, vertical integration in step 9 already provides the formalization step (formal subsumption, axioms, rules, etc.) | Viral-Hepatitis-B($x$) → [Condition($x$) $\wedge \exists y, z, w, k, m, p, f, d, h, s, a.$ Liver($y$) $\wedge$ HBVirus($z$) $\wedge$ Acute($k$) $\wedge$ Morphology($m$) $\wedge$ Process($p$) $\wedge$ Feature($f$) $\wedge$ Diagnosis($d$) $\wedge$ HBAntigen($h$) $\wedge$ Symptom($s$) $\wedge$ Patient($a$) $\wedge$ involves($x, y$) $\wedge$ inheres-in($k, x$) $\wedge$ caused-by($x, z$) $\wedge$ host($f, y$) $\wedge$ inheres-in($m, f$) $\wedge$ involves($x, p$) $\wedge$ participant($p, y$) $\wedge$ participant($p, h$) $\wedge$ part($a, y$) $\wedge$ references($d, x$) $\wedge$ conceives($a, s$) $\wedge \ldots$] |
| 11. | *Lexicalization*. Ontology data are lexicalized by re-assigning the original terms to them, and adding standard synonyms for that domain. | LEX (Viral-Hepatitis-B, {viral hepatitis, type B\|...}) LEX (HBVirus, {hepatitis B virus\|...}) etc. |
| 12. | The preliminary ontology library is refined with *context assignments* that 'modulate' concepts, axioms and terms | A research context could be interested mainly in an antigen-based view, a clinical context mainly in a patient-based view, etc. |
| 13. | Ontology data that are used in previous steps, but were not in the core or other ontologies, should be treated as in 6–13 | *Antigen* (subsumer for *HBAntigen*) and *conceives* might be lacking in the core ontology and are added to it |

in perdurants, and *regions* (spaces, usually with associated metrics) that represent the 'qualities' associated with the other components.

Situations are dependent on *descriptions* (examples of descriptions are plans, norms, theories, diagnoses, methods, recipes, etc.). Situation components have counterparts in the description layer: *courses* for activities, *functional roles* for participants, and *parameters* for regions. Description components are all taken to be *non-physical objects* that allow agents to communicate and to reason about interpretations, tasks, goals, commitments, expectations, etc. [7].

Figure 2 shows a UML diagram representing the schema for producing axioms about descriptions and situations concerning *inflammation*. In the schema, the situation components are specialized to disambiguate the multiple senses of the term *inflammation*, which can be conceptualized as a situation (a *condition*) encompassing an activity (a *biological process*), having some participants (e.g. *inflamed* tissues, antigens, antibodies) and an abstract region (a *morphology*).

Description components can also be specialized to analyse a diagnosis of inflammation. In this case, a *diagnosis* (of *inflammation*) *references* an inflammation condition, a *course* is a *path* for a biological process, *functional roles* are *played* by participants, and *parameters* are *valued* by morphologies.

### Conclusions

Conceptual tools and methodologies developed at OntoLab are being successfully used in different domains, e.g. for extracting, in a uniform way, information that is accessible only through heterogeneous systems (the semantic web being a case), as well as in building models of control systems.

A preliminary proposal could be made for the domain of molecular biology, in order to use our tools to extract and index biological information, as well as to *discover* or *verify* novel relationships across distributed data repositories.

An example of discovery or verification is an application of the 'descriptions and situations' core

Copyright © 2003 John Wiley & Sons, Ltd.

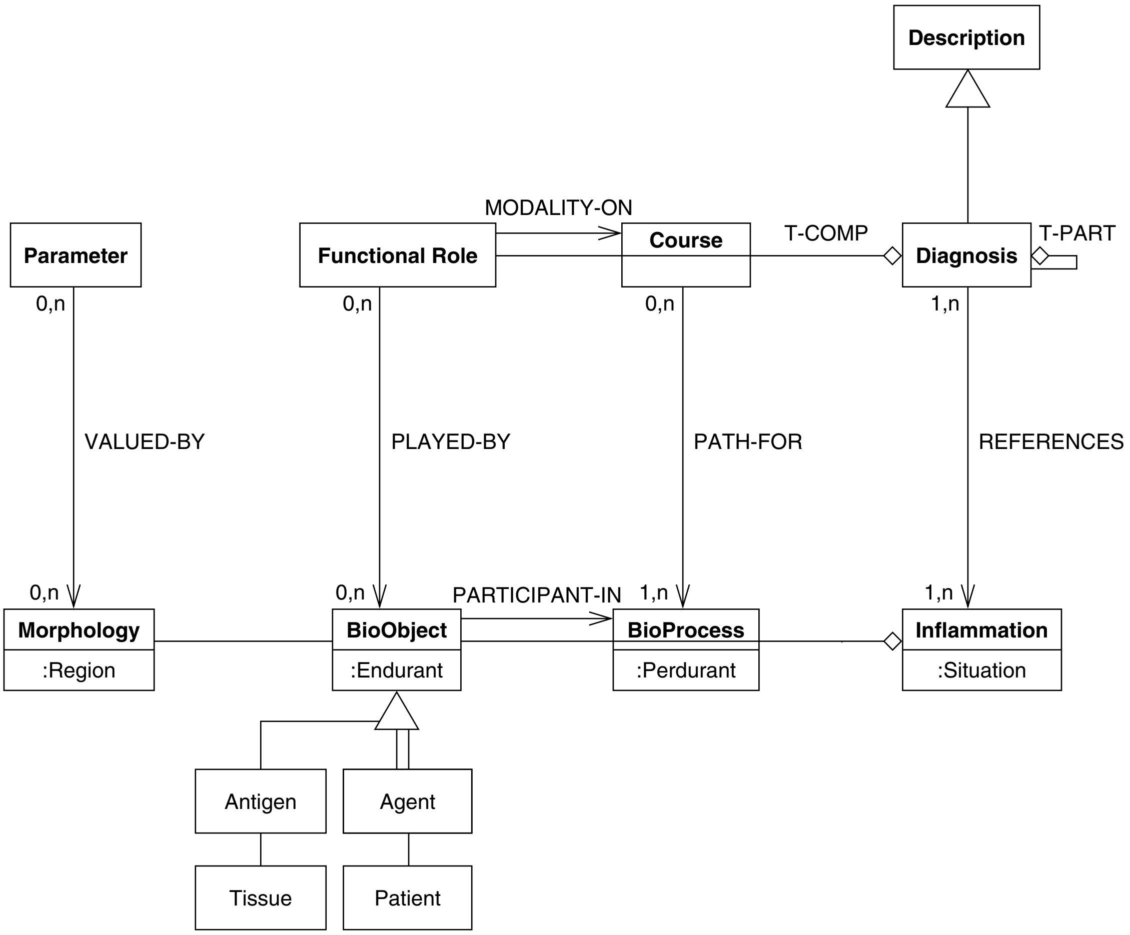

**Figure 2.** A UML class diagram for the *descriptions and situations* core axiom schema. The arrows tagged with cardinalities are relations between concepts; the rhomboidal links are part-like relations; the links with big arrows are IS_A relations axiom schema to the representation of *pathways*, e.g. if we assume that enzymes or factors are functional roles, proteins are participants in biological processes, activation values are regions, etc.

### References

1. Calvanese D, De Giacomo G, Lenzerini M. 2001. A framework for ontology integration. In *Proceedings of the 2001 International Semantic Web Working Symposium (SWWS 2001)* CA, USA.
2. Gangemi A, Pisanelli DM, Steve G. 1999. An overview of the ONIONS project: Applying ontologies to the integration of medical terminologies. *Data Knowl Eng* **31**: 183–220.
3. Gangemi A, Pisanelli DM, Steve G. 2000. Understanding systematic conceptual structures in polysemous medical terms. In *Converging Information, Technology and Health Care: Proceedings of the 2000 AMIA Annual Symposium*, Overhage JM (ed.). Los Angeles, CA, USA.
4. Gangemi A, Guarino N, Oltramari A. 2001. Conceptual analysis of lexical taxonomies: the case of WordNet top-level. In *Proceedings of the 2001 Conference on Formal Ontology and Information Systems*, Welty C, Smith B (eds). IOS Press: Amsterdam.
5. Gangemi A, Pisanelli DM, Steve G. 2001. An ontological framework to represent norm dynamics. In *Proceedings of the 2001 Jurix Conference, Workshop on Legal Ontologies*, Winkels R (ed.). University of Amsterdam.
6. Gangemi A, Fisseha F, Pettman I, *et al.* 2002. A formal ontological framework for semantic interoperability in the fishery domain. In *ECAI02 Workshop on Semantic Interoperability* Stuckenschmidt H (ed). Lyon, France.
7. Gangemi A, Pisanelli DM, Steve G. 2002. Description-Duper Ontology, Technical Report of the Laboratory for Applied Ontology. Available from: **http://ontology.ip.rm.cnr.it/Papers/TR-DDO.pdf**.
8. Guarino N, Welty C. 2002. Evaluating ontological decisions with OntoClean. *Commun ACM* **45**(2): 61–65.
9. Guarino N, Doerr M, Gangemi A 2002. OntoWeb Deliverable 3.4: harmonisation perspectives of some promising content

Copyright © 2003 John Wiley & Sons, Ltd. 

standards. Downloadable from: **http://www.ontoweb.org/download/deliverables/D3.4.pdf**.
10. Masolo C, Gangemi A, Guarino N, Oltramari A, Schneider L. 2002. WonderWeb Deliverable D17. The WonderWeb library of foundational ontologies: **http://wonderweb.semanticweb.org/deliverables/D17.shtml**.
11. Pisanelli DM, Gangemi A, Steve G. 2000. The role of ontologies for an effective and unambiguous dissemination of clinical guidelines. In *Knowledge Engineering and Knowledge Management. Methods, Models, and Tools*, Dieng R, Corby O (eds). Springer-Verlag: Berlin; 129–139.

Copyright © 2003 John Wiley & Sons, Ltd. 

